# Differential expression of several xyloglucan endotransglucosylase/hydrolase genes regulates flower opening and petal abscission in roses

**DOI:** 10.1093/aobpla/plt030

**Published:** 2013-08-02

**Authors:** Amar Pal Singh, Shveta Dubey, Deepika Lakhwani, Saurabh Prakash Pandey, Kasim Khan, Upendra Nath Dwivedi, Pravendra Nath, Aniruddha P. Sane

**Affiliations:** 1 Plant Gene Expression Laboratory, Council of Scientific and Industrial Research – National Botanical Research Institute, Lucknow 226001, India; 2 Department of Bioinformatics, CSIR-NBRI, Lucknow 226001, India; 3 Department of Biochemistry, University of Lucknow, Lucknow 226006, India

**Keywords:** Abscission, ethylene, flower opening, rose, transcriptome, xyloglucan endotransglucosylase/hydrolase.

## Abstract

Flower opening is a process that requires movement of petals from a closed position to a horizontal open position, while petal abscission requires cell-wall disassembly. Both processes are controlled by ethylene and require cell-wall modification at the junction (abscission zone) of the petal and thalamus to facilitate the movement or separation of petals. In the present study, a family of xyloglucan endotransglucosylase/hydrolase (XTH) genes was studied to understand their role in petal abscission in flowers of *Rosa bourboniana* (ethylene sensitive, early abscising) and *Rosa hybrida* (less ethylene sensitive, late abscising). Transcriptome sequencing of petal abscission zone cDNA was performed at different time points (ethylene treated and untreated) and screened for XTH genes. The study identified nine new XTH genes that showed differential changes in gene expression during flower opening and abscission. Of these, *RbXTH3*, *RbXTH5*, *RbXTH6* and *RbXTH12* were rapidly induced by ethylene within 1–4 h of ethylene treatment, corresponding to the period of flower opening. These genes also showed an early up-regulation during flower opening under ethylene-untreated (field abscission) conditions, indicating a possible role in anthesis and petal movement during flower opening. Other genes such as *RbXTH4* and *RbXTH9* were up-regulated later at 8–12 h after ethylene treatment and at 24–36 h under natural abscission conditions, indicating a possible role in abscission. Treatment with a higher ethylene dose (15 µL L^−1^ ethylene) accelerated abscission, leading to higher steady-state levels of XTH gene transcripts at an earlier time point compared with 0.5 µL L^−1^ ethylene. In contrast, transcript accumulation of most of the XTHs was considerably delayed in the late-abscising rose, *R. hybrida*, in keeping with the slower flower opening and delayed petal abscission. The results suggest coordinated action of different XTHs in cell-wall modification of xyloglucan moieties during flower opening as well as cell separation during abscission.

## Abstract (precis)

The movement of petals during flower opening (anthesis) and their separation from the parent plant during abscission requires cell wall modification at the junction (abscission zone) of the petal and thalamus. The present study shows differential ethylene mediated temporal regulation of various members of the rose XTH gene family during flower opening and abscission in the ethylene sensitive, early abscising fragrant rose and the less sensitive late abscising hybrid rose. These studies indicate that large scale changes in xyloglucan crosslinking in cell wall mediated by XTHs may facilitate movement and separation during flower opening and abscission respectively.

## Introduction

Abscission is a process of detachment of plant organs such as leaves, flowers, flower parts, fruits and seeds from the parent body. The process of separation is primarily under developmental and hormonal control, but is also strongly affected by the environment (Taylor and Whitelaw 2001; Lewis *et al.* 2006). Ethylene plays a significant role during abscission and enhances the abscission of leaves, fruits, flowers, etc., particularly in dicotyledonous plants (van Doorn 2001). Since abscission involves cell separation and dissolution of the middle lamella, the primary focus has been on some of the common cell-wall-modifying proteins and wall hydrolases such as polygalacturonases, endoglucanases, expansins, pectate lyases and pectin methyl esterases (Tucker *et al.* 1991; Kalaitzis *et al.* 1995, 1997; del Campillo and Bennett 1996; Burns *et al.* 1998; Brummell *et al.* 1999; Atkinson *et al.* 2002; Gonzalez-Carranza *et al.* 2002, 2007; Belfield *et al.* 2005; Sane *et al.* 2007; Jiang *et al.* 2008; Mishra *et al.* 2008; Sun and van Nocker 2010; Singh *et al.* 2011*a*). However, since the cell wall is a complex structure consisting of several types of wall polymers, additional players are also expected to be involved although there is not much information on these.

In recent years, the availability of complete genome sequences has allowed gene expression to be studied on a global scale using methods such as microarray and next-generation sequencing techniques. Their utilization in understanding the abscission zone (AZ) transcriptome in *Arabidopsis* stamens (Cai and Lashbrook 2008), citrus leaves (Agusti *et al.* 2008, 2009), tomato leaves (Meir *et al.* 2010) and soybean (Tucker *et al.* 2007) has helped in advancing our knowledge regarding the expression of not only cell-wall hydrolases, but also several other classes of proteins that regulate the development of the AZ and govern the progression of the process. These studies have helped in highlighting the complexity of the abscission process.

Xyloglucans are a major component of primary cell walls, accounting for up to 10–20 % of the wall component of most dicotyledonous plants (Fry 1989; Hayashi 1989). They cross-link adjacent cellulose microfibrils through non-covalent linkages, thus providing strength to the growing walls. Xyloglucan endotransglucosylase/hydrolases (XTHs) are enzymes that modify the length of xyloglucans during cell expansion through cleavage of cross-linking xyloglucan moieties (xyloglucan endohydrolase or XEH activity) and their rejoining to other xyloglucan moieties (xyloglucan endotransglucosylase or XET activity), thereby enabling the cell wall to expand without weakening (Smith and Fry 1991; Fry *et al.* 1992; Nishitani and Tominaga 1992). Xyloglucan endotransglucosylase/hydrolases belong to a large multigene family (Campbell and Braam 1999; Rose *et al.* 2002; Yokoyama *et al.* 2004), with diverse tissue-specific functions such as hydrolysis of seed storage carbohydrate (de Silva *et al.* 1993), hypocotyl elongation (Potter and Fry 1994; Catala *et al.* 1997, 2001), leaf growth and expansion (Schunmann *et al.* 1997), aerenchyma formation (Saab and Sachs 1996), fruit softening (Schroder *et al.* 1998; Ishimaru and Kobayashi 2002; Saladié *et al.* 2006), root hair initiation (Vissenberg *et al.* 2000, 2001) and tension wood formation (Nishikubo *et al.* 2007, 2011).

We previously identified two XTH genes that showed ethylene-inducible expression in petal AZs and demonstrated that abscission was associated with an increase in XET action in AZ cells (Singh *et al.* 2011*b*). In this work, we have performed a transcriptomic analysis of rose petal AZ cDNA through 454 pyrosequencing and show that a large number of XTH genes are transcriptionally active in the AZ during the course of flower opening and petal abscission.

## Methods

### Plant material and ethylene treatments

Flowers of the ethylene-sensitive, abscising rose *Rosa bourboniana* (cv Gruss an Teplitz) and the less ethylene-sensitive *Rosa hybrida*, which does not undergo abscission in the field, were chosen for study. Flowers of the same developmental stage (unpollinated, ready-to-open buds) were picked just prior to sunrise, cut with a sharp blade and the stalks immediately placed in water.

Ethylene treatment was performed in a closed airtight chamber as described by Sane *et al.* (2007) by injecting ethylene at a concentration of 0.5 µL L^−1^ for 18 h for *R. bourboniana* (time of abscission 16–18 h) or for ∼52 h for *R. hybrida* (time of abscission 48–52 h). Petal AZs (2 mm^2^ at the base of the petal in contact with the thalamus) were collected at 0 h (ethylene untreated), 1, 4, 8 and 12 h during ethylene treatment for *R. bourboniana* and additionally at 24, 36 and 48 h for *R. hybrida*. For higher ethylene doses, 15 µL L^−1^ ethylene was injected and petal AZs were collected at 0 h (untreated) and 60 min after ethylene treatment (time of abscission 3–4 h). Abscission zone tissue was immediately frozen in liquid nitrogen and transferred to −70 °C until further use. In field-abscising (natural abscission) *R. bourboniana* flowers that underwent natural pollination-induced abscission (time of abscission 38–45 h), flowers were marked at the time of opening of the outermost whorl, and petal AZs were collected at time intervals of 0, 4, 8, 12, 24 and 36 h. Abscission zones were collected and processed as above.

### RNA isolation and preparation of cDNA

RNA was isolated from frozen petal AZs of *R. bourboniana* and *R. hybrida* as described by Asif *et al.* (2000). RNA was also isolated from different tissues, namely petals, sepals, thalamus, pedicels and leaves, before ethylene treatment and after 12 h, 0.5 µL L^−1^ ethylene treatment. cDNA was prepared using the MuMLV Revertaid reverse transcriptase (Fermentas) and used for the expression analysis of different XTHs by real-time polymerase chain reaction (PCR).

### 454 pyrosequencing and identification of rose XTH genes in rose petal AZs

Total RNA from ethylene-untreated (0 h) and 0.5 µL L^−1^ ethylene-treated (8 h) petal AZs of *R. bourboniana* and *R. hybrida* was isolated along with 24 h natural AZ RNA, 8 h, 0.5 µL L^−1^ ethylene-treated petal RNA and RNA from 1 h, 15 µL L^−1^ ethylene-treated petal AZs of *R. bourboniana*. Samples were prepared and processed separately as described by Jena *et al.* (2012). For transcriptome sequencing, 5 µg of RNase-free DNase-treated total RNA was reverse transcribed for single-strand cDNA synthesis using T7 Oligo (dT) as the anchor primer. Single-stranded cDNA was further used as a template for RNase H-mediated double-strand cDNA synthesis. To enrich the amount of double-stranded cDNA, *in vitro* transcription was carried out using T7 RNA polymerase for complementary RNA (Affymetrix). Five micrograms of cRNA were reverse transcribed to single-strand cDNA using random hexamer primers, followed by RNase H-mediated double-strand cDNA synthesis. It was purified on a QIAquick PCR purification column (Qiagen) and used for cDNA library preparation as described in the manufacturer's manual (GS FLX Titanium General Library Preparation Kit, Roche 454 Company, USA). Pyrosequencing was carried out on a 454 Genome Sequencer FLX System (Roche, USA) following the standard protocol (Jarvie and Harkins 2008). In each library, >100 000 reads (40–600 bp) were generated, while the combined AZ libraries of both *R. hybrida* and *R. bourboniana* had >200 000 and >400 000 reads, respectively. Reads from different libraries were filtered and individually assembled with >90 % identity and an overlap length of 40 bp using the GS Assembler program and assembled in contigs (Huang and Madan 1999; Jena *et al.* 2012). The total transcripts in each library were queried against the TAIR and NR databases for annotation. The blastx program was used for the annotation against NR and TAIR at an *e*-value of 10^−5^. The assembled transcript sequences were analysed for putative XTH sequences in the NCBI database against all species. Nine new putative rose XTH sequences were identified [in addition to the two previously identified in our laboratory by Singh *et al.* (2011*b*)] and gene-specific primers were designed against them (after a nucleotide sequence alignment) for validation and gene expression studies such that at least the reverse primer was from the unique 3′UTR region. All the primers used in this study are given in Table 1.

**Table 1.** Sequences of primers (forward and reverse) used for real-time PCR quantification of the gene expression of various RbXTH genes.

Gene	Forward primer	Reverse primer
RbXTH3	5′AAGGATGAGATGGGTGCAGAA3′	5′TTGGTGTATGTATTGCCATGTT3′
RbXTH4	5′GGTTCAAGGATACCGGTAGGTT3′	5′AATTATGATTAACCGGCCAACAC3′
RbXTH5	5′GGATTGGGCAAGAAGGAAG3′	5′AGGTACACATGAACCAGATGAA3′
RbXTH6	5′GGTTCTAGTCTTCTAGATGACATA3′	5′CCAACAAATGAATTCAGCTCAAC3′
RbXTH7	5′AAGACACAGCAGAGTGCATGGCAGA3′	5′TGTGATCATCTGTCTTCTGGACTT3′
RbXTH9	5′CACCAGAGTGTGTGATCAATCC3′	5′AACCCTCCCTCTCATTCTCAA3′
RbXTH12	5′CCTGAGATGGGTTCAGAAGTACTTC3′	5′CCCCCCACCAAAGCTACAAA3′

### Semi-quantitative reverse transcription-polymerase chain reaction

For semi-quantitative reverse transcription–polymerase chain reaction (RT–PCR), total RNA from 8-h ethylene-untreated and ethylene-treated tissues such as sepals, petals, thalamus, pedicels and leaves was isolated and treated with DNase I (Fermentas) and purified. For cDNA preparation, 5 µg of purified total RNA from each sample were reverse transcribed using 3′AP and Revertaid reverse transcriptase (Fermentas). An equal quantity (25–30 ng) of cDNA was used for PCR amplification of the target transcript. Rose *β-ACTIN* was used as an internal control and PCR was carried out in an Eppendorf PCR machine (Mastercycler Epgradient S, Germany).

### Real-time RT–PCR

For real-time PCR, rose *β-ACTIN* was used as an internal control (Singh *et al.* 2011*b*). The real-time reaction was carried out using the SYBR Green Dye master mix (Fermentas) in triplicate (technical replicates) for each sample consisting of pooled AZs from a given time point (collected over a month) on an ABI 7500 real-time PCR machine (Applied Biosystems Inc., USA). The data analysed were the mean of triplicates. For *RbXTH3* and *RbXTH6*, where a high but transient ethylene-induced expression was observed for 0.5 µL L^−1^ ethylene-treated AZ cDNA, the data were repeated on a biological replicate. The general steps performed during real-time PCR experiments were as follows: step 1, 50 °C for 2 min; step 2, 95 °C for 10 min; step 3, 95 °C for 15 s and 60 °C for 1 min, ×40 cycles. The *C_T_* values obtained from the internal control (actin) and experimental samples were plotted on an Excel sheet and Δ*C_T_* values were calculated. The Δ*C_T_* value was used to calculate ΔΔ*C_T_* and the fold change in expression was calculated by the 2^−ΔΔ*C_T_*^ method (Livak and Schmittgen 2001). Relative change in the mRNA expression was calculated for each gene by normalizing the *C_T_* values against the internal control rose β-actin and expressed as a ratio against the averaged value of 0 h which was considered as one.

### Sequence alignment and phylogenetic analysis

Amino acid sequence alignment of the different RbXTHs identified from transcriptome sequencing of rose AZ cDNA was carried out using CLUSTAL W. The sequences of RbXTH1 and RbXTH2 (Singh *et al.* 2011*b*) were also included along with those of *Populus* XET16A (as an example of a protein showing XTH activity) and nasturtium (*Tropaeolum majus*) TmNXG1 (as an example of a protein showing XEH activity) (Baumann *et al.* 2007). For the dendrogram, only complete polypeptide sequences of rose XTHs (RbXTH1 to RbXTH6 and RbXTH12) were chosen along with *Arabidopsis* XTHs, *Populus* XET16A and *T. majus* TmNXG1. Analysis was carried out using the MEGA 5 software (Tamura *et al.* 2011). The amino acid sequences were first aligned by CLUSTAL W and then a UPGMA (Unweighted Pair Group Method with Arithmetic mean) tree was constructed by the bootstrap method.

## Results

### Isolation of nine new XTH genes from rose AZ cDNA

The total transcriptome consisting of data obtained separately from different AZ cDNA samples of *R. bourboniana* and *R. hybrida* was organized into contigs, and these contigs as well as singletons were then screened against the NCBI database for identification of putative XTH sequences. A total of 11 XTH sequences were identified, of which two, *RbXTH1* and *RbXTH2*, had been previously characterized (Singh *et al.* 2011*b*). The rest were named as *RbXTH3* to *RbXTH12*. Of these, five, namely *RbXTH3*, *RbXTH4*, *RbXTH5*, *RbXTH6* and *RbXTH12*, included the complete open reading frames. The others, namely *RbXTH7*, *RbXTH8*, *RbXTH9* and *RbXTH10*, were partial sequences lacking the 5′ end which coded for the N-terminal end. An alignment of the predicted polypeptide sequences was performed along with *Populus* PtXET16 (known to function as an XET) and TmNXG1 (known to function as a xyloglucan endotranshydrolase) (Baumann *et al.* 2007). As shown in Fig. 1A, essential regions within the XTH proteins, namely DEI/LDF/IEFLG, were conserved in all the proteins (except for RbXTH10, which was a partial sequence and lacked this region). Interestingly, the glycosylation site NXT/S adjacent to the catalytic site could be seen in seven sequences (including RbXTH1 and RbXTH2) but was absent in RbXTH4, RbXTH5 and RbXTH9. The latter three sequences showed greater similarity to the TmNXG1 sequence, which also lacks this glycosylation site. Although the DYNII extension in TmNXG1, shown to be necessary for xyloglucan endohydrolase activity (XEH; Baumann *et al.* 2007), was missing in RbXTH4, 5 and 9, some similarity in the flanking portion (viz. the presence of GR residues following DYNII) could be observed in this region in these polypeptides but not in the others.

**Figure 1.**
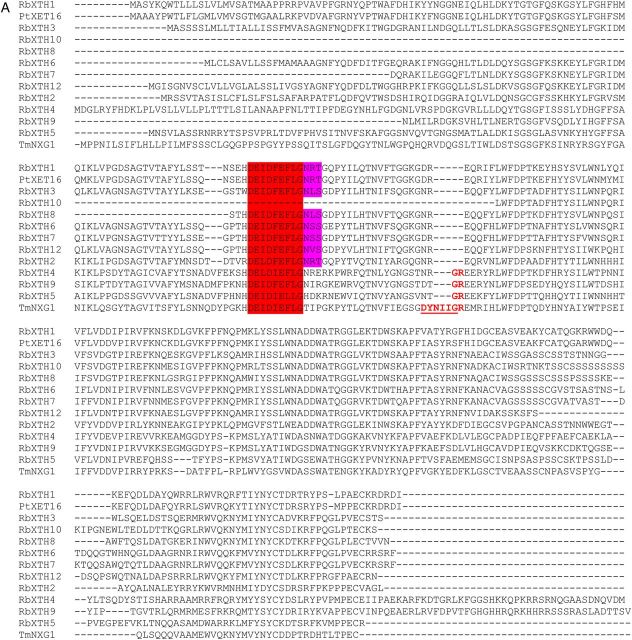
(A) CLUSTAL W alignment of the predicted amino acid sequences of different RbXTHs identified from transcriptome sequencing of rose AZ cDNA. The sequences of RbXTH1 and RbXTH2 were also included along with those of *Populus* XET16A and nasturtium (*T. majus*) TmNXG1. The conserved catalytic site DEI/LDF/IEFLG is highlighted in dark red. The *N*-glycosylation site (NxS/T) is shown in violet. The DYNII extension in TmNXG1, responsible for XEH activity as well as the GR residues (conserved between XTH4, XTH5, XTH9 and TmNXG1) are shown in red.

**Figure 1 continued.**
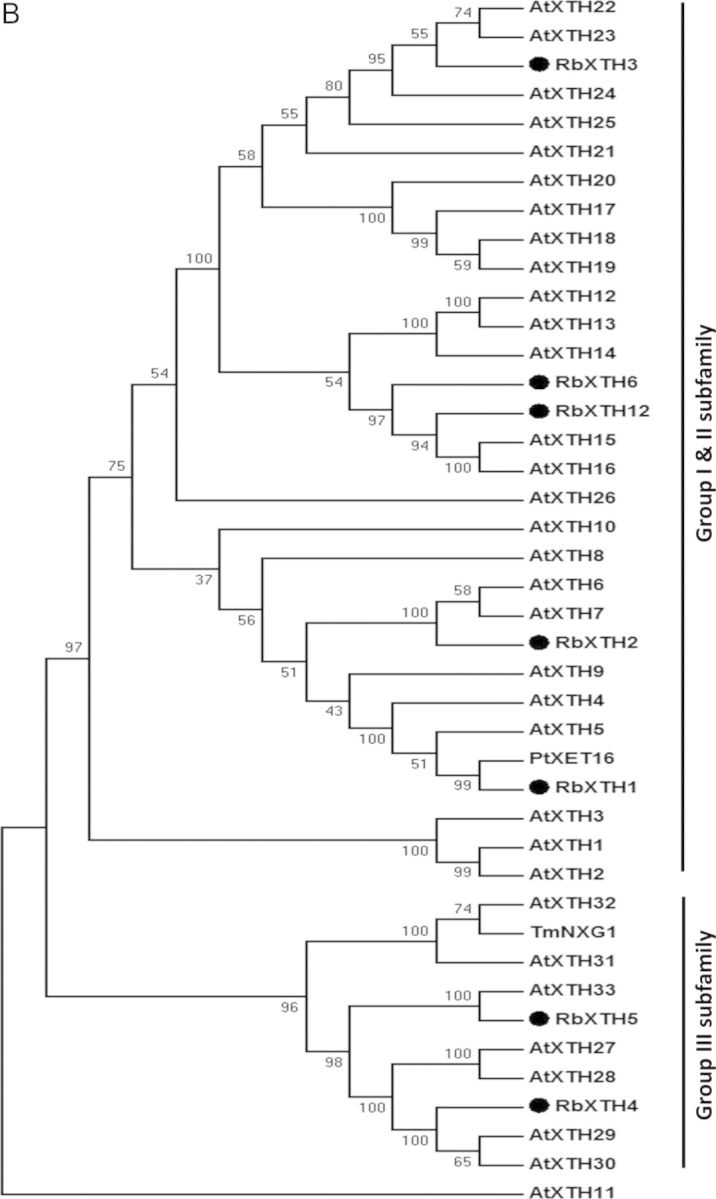
(B) Phylogenetic analysis of the full-length rose XTH amino acid sequences with *Arabidopsis* XTHs, *Populus* XET16A and nasturtium (*T. majus*) TmNXG1 sequences. Analysis was carried out using the MEGA 5 software. The amino acid sequences were aligned by CLUSTAL W and the UPGMA dendrogram was constructed by the bootstrap method.

A phylogenetic analysis of the seven full-length RbXTHs (including RbXTH1 and RbXTH2) was carried out using MEGA5 with PtXTH16 and TmNXG1 and all the *Arabidopsis* XTH sequences. As shown in Fig. 1B and reported previously by Yokoyama *et al.* (2004), the majority of the XTHs in *Arabidopsis* (classified as Group I and Group II) clustered together. RbXTH1 and RbXTH2 appeared in one subclade (with RbXTH1 appearing closest to PtXET16), while RbXTH3 and RbXTH6 (along with RbXTH12) appeared in separate subclades of the combined Group I/II. RbXTH4 and RbXTH5 appeared in a separate group (designated as Group III) that included TmNXG1. Some of the Group III members, such as AtXTH31 and AtXTH32 (besides TmNXG1), have been shown to function as XEHs (Kaewthai *et al.* 2013).

### Expression profiling of rose *XTH* genes during petal abscission

**Figure 2.**
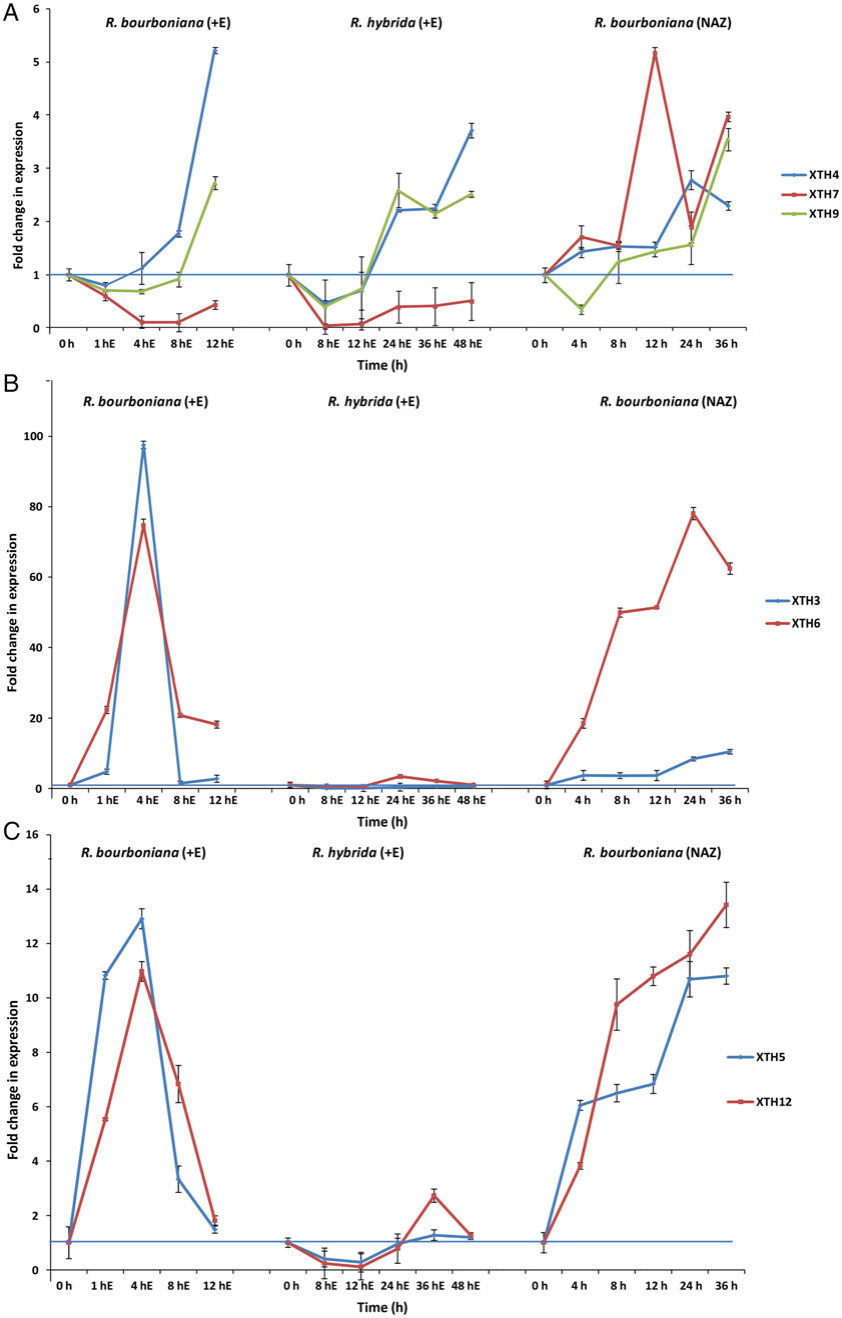
(A) Real-time PCR analysis of *RbXTH4*, *RbXTH7* and *RbXTH9* transcript accumulation in petal AZs of 0.5 µL L^−1^ ethylene-treated flowers of *R. bourboniana* and *R. hybrida* and in flowers of *R. bourboniana* undergoing natural abscission (NAZ). Total RNA from petal AZs was isolated from the 0-h, ethylene-untreated sample (control) as well as from samples treated with ethylene. Reactions were run in triplicate from a pool of AZ RNA samples harvested from at least 50 flowers. The averaged value of the 0-h (ethylene untreated, control) AZ sample was considered as 1 and the averaged values of the other samples were calculated against the 0-h sample. Rose *β-ACTIN* was used as an internal control for normalization. (B) Real-time PCR analysis of *RbXTH3* and *RbXTH6* transcript accumulation in petal AZs of 0.5 µL L^−1^ ethylene-treated flowers of *R. bourboniana* and *R. hybrida* and in the flowers of *R. bourboniana* undergoing natural abscission. Analysis was carried out as described in (A). (C) Real-time PCR analysis of *RbXTH5* and *RbXTH12* transcript accumulation in petal AZs of 0.5 µL L^−1^ ethylene-treated flowers of *R. bourboniana* and *R. hybrida* and in the flowers of *R. bourboniana* undergoing natural abscission. Analysis was carried out as described in (A).

To understand the regulation of the different XTH genes identified through the transcriptome sequencing, a time course study of transcript accumulation of various rose XTH genes was carried out by real-time RT–PCR in 0.5 µL L^−1^ ethylene-treated petal AZs of *R. bourboniana* and *R. hybrida* as well as under conditions of natural abscission. As shown in Fig. 2A, transcripts of *RbXTH4* began to increase from 8 h of ethylene treatment, reaching a maximum (∼3- to 5-fold of the control) at 12 h of ethylene treatment just prior to abscission. *RbXTH9* did not show much of a change until 8 h but increased at 12 h. In *R. hybrida*, a similar expression pattern for these two genes was observed during abscission but with a delay so that accumulation occurred late, from 24 h onwards, with the peak of transcript accumulation being attained at 48 h (just prior to abscission). In contrast to these genes, *RbXTH7* showed a down-regulation in transcript levels during the course of abscission in both *R. bourboniana* and *R. hybrida*. Under conditions of natural (field) abscission, there was a gradual increase in transcript accumulation but with a delay in the maximum transcript accumulation that occurred at 24 h for *RbXTH4* and 36 h for *RbXTH9*. This corresponded with the delay in abscission occurring under these conditions. Surprisingly, *RbXTH7*, which was repressed during ethylene-induced abscission, showed a substantial increase in transcript accumulation during natural abscission.

In contrast to the relatively late accumulation of the above-mentioned genes, *RbXTH3* and *RbXTH6* showed a rapid and very high but transient transcript accumulation in ethylene-treated *R. bourboniana* petal AZs (Fig. 2B). The relative transcript levels of *RbXTH3* and *RbXTH6* increased by ∼100- and 75-fold, respectively, within 4 h of ethylene treatment as against the 0-h sample. After this, the transcript levels declined rapidly at 8 h/12 h, although they still showed a 20-fold increase in *RbXTH6* compared with the 0-h sample. Unlike in *R. bourboniana*, petal abscission in *R. hybrida* was not associated with any change in the transcript levels of *RbXTH3* but with only a small change in *RbXTH6* (which occurred at the 24-h stage). Interestingly, under field conditions, *RbXTH3* showed a gradual increase during abscission up to 36 h, while *RbXTH6* showed a substantial increase as early as 4 h onwards right up to 24 h with only a slight decrease at 36 h.

The third set of genes shown in Fig. 2C included *RbXTH5* and *RbXTH12*, which were also rapidly up-regulated within 1–4 h of ethylene treatment (although not to the extent of *RbXTH3* and *RbXTH6*) before showing a decline. In *R. hybrida*, only a marginal increase in the transcript level of *RbXTH12* was observed at 36–48 h. No significant change was observed in *RbXTH5*. Interestingly, both genes showed a rapid and substantial increase in transcript accumulation during the entire course of natural petal abscission right from the 4-h time point onwards, with peak values at 36 h.

Two genes, *RbXTH8* and *RbXTH10*, could not be studied due to difficulties in amplification.

### Transcript accumulation under high-dose ethylene treatment

**Figure 3.**
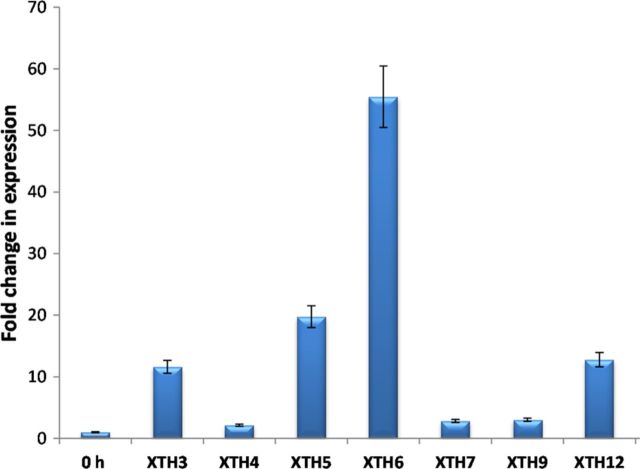
Real-time PCR analysis of *RbXTH* gene transcript accumulation in petal AZs of 1-h high-dose (15 µL L^−1^) ethylene-treated flowers of *R. bourboniana* compared against the ethylene-untreated control AZ sample (0 h). The expression of each gene was measured at 0 h and at the 1-h high-dose ethylene time point. The relative expression at 0 h for each gene was considered as 1 (and is depicted as ‘0-h’ expression) and the relative expression of the gene of interest at the 1-h time point was calculated accordingly as described in Fig. 2A.

Previous studies in our laboratory had shown that abscission of rose petals in *R. bourboniana* can be accelerated by treatment with a high ethylene dose (15 µL L^−1^) with abscission occurring within 3–4 h of ethylene treatment (Singh *et al.* 2011*a*, *b*). In order to test whether acceleration of abscission by 15 µL L^−1^ ethylene was associated with a more rapid up-regulation of the different XTH genes, transcript accumulation of these genes was studied following 1 h of 15 µL L^−1^ ethylene. As shown in Fig. 3, almost all the genes showed rapid transcript accumulation at the 1-h stage. Genes such as *RbXTH4*, *RbXTH7* and *RbXTH9*, which were not detectable at the 1-h time point in 0.5 µL L^−1^ ethylene-treated samples, showed a 3- to 4-fold increase in transcript levels 1 h after 15 µL L^−1^ ethylene treatment. Even among the early ethylene-responsive genes such as *RbXTH3*, *RbXTH5*, *RbXTH6* and *RbXTH12*, the transcript accumulation after 1 h, 15 µL L^−1^ ethylene was higher than that after 0.5 µL L^−1^ treatment.

### Expression of rose *XTH* genes in different tissues in response to ethylene

**Figure 4.**
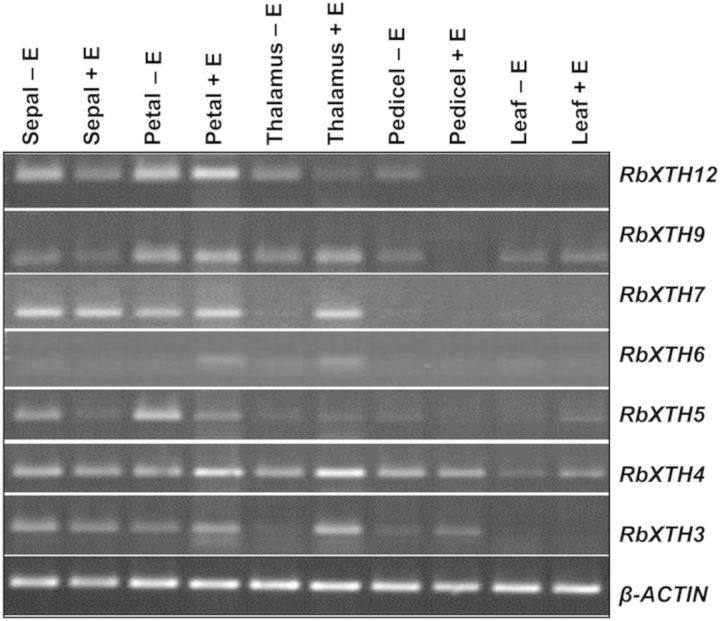
Comparative transcript accumulation of different *RbXTH* genes in ethylene-untreated and ethylene-treated tissues of *R. bourboniana* as determined by semi-quantitative RT–PCR. Cut rose flowers were treated with or without 0.5 µL L^−1^ ethylene for 8 h. *β-ACTIN* was used as an internal control.

To understand the tissue specificity and ethylene inducibility of the rose *XTH* genes identified, semi-quantitative RT–PCR was performed with rose *β-ACTIN* as an internal control. Expression was tested in both ethylene-untreated and 8-h, 0.5 µL L^−1^ ethylene-treated tissues such as sepals, petals, thalamus, pedicels and leaves. As shown in Fig. 4, most of the genes were transcribed to varying levels in all tissues studied. Of these, *RbXTH3*, *RbXTH4*, *RbXTH6* and *RbXTH7* showed a clear ethylene-responsive transcriptional up-regulation in the thalamus and, to a lesser extent, in petals. For genes such as *RbXTH3*, *6* and *7*, the transcriptional up-regulation in the thalamus was quite prominent with barely any transcript visible in ethylene-untreated samples. Interestingly, ethylene-responsive up-regulation could not be observed for any of the genes in sepals and only weakly for *RbXTH4* and *RbXTH5* in leaves. Ethylene repressed the transcription of *RbXTH5*, *RbXTH9* and *RbXTH12* in sepals and pedicels, and also in the thalamus for *RbXTH12* and in petals for *RbXTH5*. These results indicated tissue-specific control over regulation of the genes in response to ethylene.

## Discussion

Xyloglucans are an important component of dicotyledonous cell walls, accounting for up to 20 % of the cell wall (Fry 1989; Hayashi 1989). Thus, it is not surprising that plant genomes contain a large number of XTH genes with up to 33 genes in *Arabidopsis* (Yokoyama and Nishitani 2001), 29 in rice (Yokoyama *et al.* 2004) and 41 in *Populus* (Geisler-Lee *et al.* 2006). Although these genes have been shown to have diverse expression patterns during plant development (Yokoyama *et al.* 2004), their involvement in organ abscission has only recently been observed through large-scale gene expression studies in *Arabidopsis* (Lashbrook and Cai 2008), citrus (Agusti *et al.* 2009) and tomato (Meir *et al.* 2010). Our studies have led to the identification and characterization of nine hitherto unknown XTH genes in abscission through 454 pyrosequencing in addition to the two (*RbXTH1* and *RbXTH2*) previously identified (Singh *et al.* 2011*b*). This is a considerably larger number than reported previously in AZs in *Arabidopsis*, citrus or tomato, indicating that abscission in rose petals may proceed through large-scale modifications in xyloglucan cross-linking. Some of these genes, such as *RbXTH3*, *RbXTH5*, *RbXTH6* and *RbXTH12*, are rapidly up-regulated within 1–4 h of ethylene treatment in *R. bourboniana* and, except for *RbXTH3*, remain quite high until 8 h. Under our conditions, this early period from 0 to 4 h corresponds to anthesis when flowers open and petals unfurl. Ethylene has been shown to facilitate flower opening in roses and other flowers (Reid *et al.* 1989; Yamamoto *et al.* 1994; Ma *et al.* 2006; Xue *et al.* 2008), and this opening can be partly blocked by 1-methyl cyclopropene. Flower opening is associated with petal expansion and expression of several expansins and XTHs (Evans and Reid 1988; Yamada *et al.* 2009; Harada *et al.* 2011). In addition to cell-wall expansion of petals, movement of the petals from a vertically upright position (at the 0 stage buds) to a horizontal position (4 h open stage) would have to involve changes in the cell wall at the point of attachment of the petals to the thalamus, i.e. at the AZ, so as to allow the petals to change position and unfurl. In this respect, it is interesting to note that the ethylene-induced AZ-related expression of *RbXTH3*, *RbXTH5*, *RbXTH6* and *RbXTH12* appears much higher than their expression in the adjacent ethylene-sensitive tissues such as the thalamus and petals. It is likely that the ethylene-mediated up-regulation of these four genes might facilitate rapid changes in the cross-linking xyloglucan moieties in the AZ, leading to a greater flexibility of the cell wall, which in turn might allow the petals to move from an upright position to an open horizontal position. Thus, these are more likely to represent genes that are required for flower opening rather than abscission. Indeed, the high expression level of most of these genes earlier during the course of natural abscission (where abscission is delayed) also suggests a role in flower opening rather than abscission. Of these, *RbXTH6* is expressed predominantly in petal AZs with very little expression in other tissues, suggesting a specific role in flower opening. Most of these genes show very little expression in non-reproductive tissues such as leaves and pedicels. Other genes such as *RbXTH4* and *RbXTH9* appear later, from 8 to 12 h in ethylene-treated flowers and much later during natural abscission, similar to what has been observed previously for *RbXTH1* and *RbXTH2* (Singh *et al.* 2011*b*). Since they are up-regulated just prior to abscission, these are more likely to represent genes that are activated in response to abscission cues. Such temporal differences in transcription have also been observed in *Arabidopsis* stamen abscission for XTHs as well as other wall hydrolase genes (Lashbrook and Cai 2008). Treatment with 15 µL L^−1^ ethylene accelerates abscission to just 3 h with abscission occurring without complete petal opening. This is associated with a much earlier (and higher) transcript accumulation of most XTH genes than when treated with 0.5 µL L^−1^ ethylene, suggesting a strong correlation between ethylene, XTH gene activation and cell separation in the AZ.

Interestingly, the same dose of ethylene that activates the XTH genes within 1–8 h in *R. bourboniana* leads to activation of only some of these genes in *R. hybrida* and that too after a delay of ∼16–24 h (or more). The inability of these genes to get activated rapidly even when the flowers are exposed to exogenous ethylene indicates that some components of ethylene perception or signalling may be affected in *R. hybrida*, leading to a slower and delayed response in these plants. Although one cannot rule out a change in promoter sequences of these genes between *R. bourboniana* and *R. hybrida*, it seems rather unlikely that promoters of all XTH genes in *R. hybrida* would have undergone a change that makes them relatively insensitive to ethylene.

Of all the genes studied, *RbXTH7* shows a different expression pattern in that it is actually down-regulated by ethylene in *R. bourboniana* and *R. hybrida* but up-regulated from the 4-h time point under conditions of natural abscission. The expression pattern appears to indicate an ethylene-induced repression of *RbXTH7*. This does not explain the high expression levels under natural conditions but it does suggest that regulatory controls under natural field conditions (where the flowers are still attached to the parent body) and under excised ethylene-induced conditions may be different, leading to a change in expression patterns.

We have previously shown that transcription of the XTH genes is associated with an increase in XET activity as determined by *in situ* co-localization of fluorescent sulforhodamine-conjugated xyloglucan oligosaccharides in the AZ (Singh *et al.* 2011*b*). Our studies had revealed a strong fluorescence in the 8-h ethylene-treated petal AZs and the 24-h natural AZ samples. The present study shows that this increase in XET activity is likely to be a consequence of not just RbXTH1 and RbXTH2 functions but also from the cumulative action of most of the XTHs identified from these two studies. However, while transglucosylase action had clearly been demonstrated by Singh *et al.* (2011*b*), the presence of xyloglucan endohydrolase action is still unclear. Three of the proteins, viz. RbXTH4, RbXTH5 and RbXTH9, do show some domain similarity to TmNXG1 and are clustered in the same group, and might well function to aid abscission via hydrolysis of the xyloglucan moieties. However, these would require further enzymatic studies using expressed proteins of RbXTH4, RbXTH5 and RbXTH9.

## Conclusions

This study shows that petal abscission in rose is associated with transcriptional up-regulation of a large repertoire of XTH genes that most likely aids both movement during flower opening as well as petal abscission through complex tissue-specific ethylene-responsive expression of the genes.

## Accession Numbers

RbXTH3-JX259257

RbXTH4-JX259258

RbXTH5-JX259259

RbXTH6-JX259260

RbXTH7-JX259261

RbXTH8-JX259262

RbXTH9-JX259263

RbXTH10-JX259264

RbXTH12-JX259265

## Sources of Funding

This work was supported by funds from the Department of Biotechnology, Government of India, New Delhi, from a network project (BSC0107) from the Council of Scientific and Industrial Research (CSIR), India and by CSIR Senior Research Fellowships to A.P.S. and S.P.P.

## Contributions by the Authors

A.P.S. carried out the transcriptome work and A.P.S., S.D. and S.P.P. performed the RT–PCR analysis. K.K. performed the phylogenetic analysis, while D.L. carried out the assembly and bioinformatic analysis of the transcriptome for identification for XTHs. U.N.D., P.N. and A.P.S. discussed the results. A.P.S. planned the work and wrote the paper.

## Conflict of Interest Statement

None declared.
